# Physicians’ Use of Electronic Health Record Data Elements and Decision Support Tools in Heart Failure Management: User-Centered Cross-Sectional Survey Study

**DOI:** 10.2196/79239

**Published:** 2025-11-14

**Authors:** Mohamed S Ali, Bruna Oewel, Kaitlyn M Greer, Sabah Ganai, Mark W Newman, Kelly Murdoch-Kitt, Scott L Hummel, Michael P Dorsch

**Affiliations:** 1 College of Pharmacy, University of Michigan, 428 Church Street, Ann Arbor, MI, 48109, United States, 1 734-764-7312; 2 Penny W. Stamps School of Art & Design, University of Michigan, Ann Arbor, MI, United States; 3 School of Information, University of Michigan, Ann Arbor, MI, United States; 4 Medical School, University of Michigan, Ann Arbor, MI, United States; 5 Frankel Cardiovascular Center, University of Michigan, Ann Arbor, MI, United States; 6 VA Ann Arbor Healthcare System, Ann Arbor, MI, United States

**Keywords:** heart failure management, electronic health records, clinical decision support, cardiologists, general medicine physicians, workflow integration, health care technology, predictive models, provider satisfaction, user-centered design, user interface design, user experience design, health informatics

## Abstract

**Background:**

The management of heart failure (HF) requires complex, data-driven decision-making. Although electronic health record (EHR) systems and clinical decision support (CDS) tools can streamline access to essential clinical information, it remains unclear which EHR elements and tools cardiologists and general medicine physicians prioritize when caring for patients with HF.

**Objective:**

This study aims to identify these elements and tools to improve the user interface design of future EHR applications.

**Methods:**

This study used a user-centered design research approach to understand physician workflows and decision-making needs in HF care. A cross-sectional online survey was administered to 302 physicians, comprising 150 cardiologists (including 15 HF specialists) and 152 general medicine physicians. Respondents reported their use of EHR variables (eg, medication lists, laboratory results, diagnostic tests, problem lists, clinical notes) for decision-making in HF care, as well as their time spent in the EHR before, during, and after patient visits along with their use of predictive models and patient-reported outcome questionnaire. Descriptive analyses, *χ*^2^ tests, and *t* tests were conducted to compare groups, with statistical significance set at *P*<.05.

**Results:**

A total of 302 health care providers participated in the survey, nearly evenly split between cardiologists (49.7%, 150/302) and general medicine physicians (50.3%, 152/302). Both groups consistently relied on medication lists, vital signs, laboratory results, diagnostic tests, problem lists, and clinical notes for HF decision-making. Cardiologists placed greater emphasis on diagnostic tests for inpatient HF care (mean [SD] overall frequency, 4.66 [0.50] vs 4.44 [0.64]; *P*=.012) and outpatient HF care (mean [SD] overall frequency, 4.67 [0.55] vs 4.35 [0.71], *P*<.001). In contrast, general medicine physicians relied more on problem lists for inpatient HF care (mean [SD] overall frequency, 4.63 [0.58] vs 4.43 [0.72], *P*=.034), with no significant difference in the outpatient setting (*P*>.05). Both groups underutilized standardized questionnaires and predictive models, with only 20.1% (29/144) of cardiologists and 4.5% (6/133) of general medicine physicians using standardized questionnaires (*P*<.001)

**Conclusions:**

Both physician groups depend on medication lists, laboratory results, diagnostic tests, and problem lists. Cardiologists prioritize diagnostic tests, whereas general medicine physicians more often use problem lists. Low use of questionnaires and predictive models highlights the need for better integration of these tools. Future EHR design interface should tailor functionalities to accommodate these differing priorities and optimize HF care.

## Introduction

### Background

Heart failure (HF) affects more than 64 million people globally [1], placing a significant burden on health care systems [2,3]. In the United States, HF is a leading cause of hospitalization among older adults [4] and is associated with high rates of morbidity and mortality [2,3]. Effective management of HF requires timely and accurate decision-making based on multiple clinical variables, including patient history, laboratory results, imaging, and treatment adherence [5-7]. Ensuring that this critical information is readily available to health care providers in a clear and timely manner is a crucial step in achieving optimal HF management.

An electronic health record (EHR) is an electronic version of a patient’s medical history that is maintained by the health care provider over time [8]. It includes all key administrative and clinical data relevant to the patient’s care under that provider, such as demographics, progress notes, problem lists, medications, vital signs, past medical history, immunizations, laboratory results, and radiology reports [8,9]. By automating access to information, the EHR has the potential to streamline clinical workflows and make clinical information readily available to health care providers [8,10]. It can also support a range of care-related activities—either directly or indirectly—through interfaces designed for evidence-based decision support [10].

A clinical decision support system (CDSS) delivers timely information, usually at the point of care, to assist health care providers in making informed decisions about a patient’s care [11]. When integrated with an EHR, a CDSS can access relevant patient data, highlight key clinical information, and provide tailored recommendations to health care providers [12].

### Knowledge Gap

The first step in designing a new health information technology system for CDSS is to identify the needs of users and define the system’s intended functions. A design process includes and revolves around communication with end-users to ascertain their behaviors, motivations, pain points, and needs, as a user-centered design. A user-centered design is an approach for developing applications that incorporates user-centered activities throughout the entire development process [13]. This approach enables end-users to shape the design, enhancing overall usability [14]. In HF, decision-making largely depends on clinical variables and tools, especially when initiating or titrating medications [5-7]. However, identifying the specific EHR variables and tools that cardiologists and general medicine physicians prioritize during HF management remains essential. While prior work has characterized general specialty differences in EHR use [15], the limited uptake of risk prediction tools [16], and patient-reported outcomes in routine practice [17], no study has, for HF specifically, quantified which EHR data elements clinicians deem most important, nor contrasted cardiology versus general medicine priorities across inpatient and outpatient care.

### Objective

Accordingly, this study aimed to determine which EHR information and tools these providers consider most important in their clinical decision-making while caring for patients with HF. Consequently, future CDSSs can be designed and developed accordingly.

## Methods

### Study Design

We used a mixed methods, user-centered approach that incorporated a variety of quantitative and qualitative techniques. This study was a cross-sectional survey of a diverse pool of physicians. We recruited a group of cardiologists, HF specialists, and general medicine physicians through Dynata (Dynata, Shelton, CT, USA), a large data firm that maintains survey participant panels, to answer clinical scenario questions in an online survey. The anonymous online survey was developed using Qualtrics. We stopped the survey once we reached 302 physicians (150 cardiologists and 152 general medicine physicians). Because Dynata recruits participants until a predefined quota is achieved, the response rate information is not available for this study.

### Survey Instrument

The survey was developed and informed based on insights from earlier phases of user-centered research to reduce provider workload, enhance provider decision-making, and improve patient care. This included provider (physician, nurse, pharmacist, or physician assistant) observations, interviews, and prototyping at the University of Michigan, the Veterans Affairs Ann Arbor Healthcare System**,** St. Joseph Mercy Ann Arbor Hospital, and Henry Ford Hospital and a review of existing literature on EHR-based CDSS for HF management. Specific areas observed included the tools the EHR providers use both inside and outside to care for patients with HF, the pain points encountered when using the EHR system to manage patients with HF, the time required to review a patient in the EHR, and the tools used to streamline workflow in patient management.

The questionnaire (Multimedia Appendix 1) consisted of several domains, including the provider’s clinical role, whether they treat patients with HF, the percentage of time they spend on HF care, and the types of patients they manage (inpatients, outpatients, or both). It also gathered information on years of experience using EHRs, the amount of time spent interacting with the EHR before, during, and after patient visits, the number of software applications and predictive tools used daily, and the most frequently used EHR vendor. Most importantly, the survey included a Likert-scale question assessing how frequently providers rely on a list of clinical information for HF treatment decisions in both inpatient and outpatient settings. Participants were recruited from a national online physician panel (Dynata). The survey did not implement stratified sampling by geographic region, institutional type (eg, academic vs community), or practice ownership. To enable planned comparisons, the survey used a quota by specialty (cardiology vs general medicine); otherwise, enrollment was consecutive until the target sample size was reached. The study reported practice setting (inpatient, outpatient, or both), EHR vendor, and years of EHR use to characterize sample diversity. The survey was validated through an independent expert review by HF cardiologists, HF clinical pharmacists, and survey methodologists; we incorporated their feedback via iterative revisions to improve clarity, relevance, and completeness.

### Statistical Analysis

Descriptive statistics were used to summarize the baseline characteristics of the participants, which included their clinical roles, the time providers spent caring for patients with HF, the EHR systems used, and the duration of EHR usage before, during, and after patient visits. Frequencies and percentages were calculated for each category.

Our primary variable of interest was the clinical variables that providers used in clinical decision-making for HF and how these were prioritized. Responses to survey questions related to our primary variable of interest were captured using a 5-point Likert scale. The scale ranged from 1 to 5, where 1=never, 2=rarely, 3=sometimes, 4=often, and 5=always. Providers were asked to indicate how frequently they used that information in their decision-making process for each type of Likert-scaled clinical information (eg, diagnostic tests, laboratory results, and clinical notes). The responses were numerically coded according to the Likert scale, and the mean score for each group (cardiologists and general medicine physicians) was calculated by averaging the numerical responses. The SD was also calculated to indicate the variability in responses within each variable. Our secondary variable of interest was the time spent on EHRs before, during, and after patient visits, as well as the number of software systems used daily by both cardiologists and general medicine physicians.

The *χ*^2^ tests were used to assess differences in categorical variables for comparison between provider groups (eg, cardiologists vs general medicine physicians). The *t* tests were used for continuous variables, with statistical significance set at *P*<.05. R programming (version 4.4.2; R Foundation for Statistical Computing) and SAS (version 9.4; SAS Institute Inc.) were used to conduct these analyses and generate the figures.

### Ethical Considerations

This study was deemed exempt by the University of Michigan Institutional Review Board because it was not found to constitute human subject research. All participants provided electronic informed consent within Qualtrics before beginning the survey. Recruitment and compensation were managed by Dynata; participants received panel-standard incentives, and the study team did not access personal contact information or payment details.

## Results

### Participants

A total of 302 health care providers participated in the survey with cardiologists representing 49.7% (150/302) of the population and general medicine physicians representing 50.3% (152/302) of the population. Of the general medicine group, 50% (76/152) were family medicine physicians and 50% (76/152) were internal medicine physicians. Among cardiologists, 10% (15/150) specialized in HF. In terms of time spent managing HF, 42.7% (129/302) of respondents spent 1%‐24% of their time, 37.4% (113/302) spent 25%‐49%, 10.3% (31/302) spent 50%‐74%, and 9.6% (29/302) spent 75%‐100%. Most (66.6%, 197/302) provided both inpatient and outpatient care; 27% (80/302) cared only for outpatients, and 6.4% (19/302) only for inpatients.

### EHR Use Across the Visit Workflow

EHR usage varied across the clinical workflow. Before visits, 58% of cardiologists (87/150) and 64% of general medicine physicians (97/152) reported spending 3‐10 minutes in the EHR, whereas during visits, usage was typically between 1 and 5 minutes. After visits, 61% of the respondents (184/302) reported 3‐10 minutes of use. Overall, 92.4% of participants (279/302) had over 5 years of experience with EHRs. Detailed distributions are provided in Table 1. The time spent reviewing EHR before, during, and after patients’ visits is shown in Table 2.

**Table 1.** Baseline provider demographics.

Variable	Value, n (%; n=302)
Cardiologist	150 (49.7)
HF Cardiologist	15 (10.0)
General Medicine	152 (50.3)
Family Medicine	76 (50.0)
Internal Medicine	76 (50.0)
Percent of time the provider spent caring for patients with heart failure	
1%‐24%	129 (42.7)
25%‐49%	113 (37.4)
50%‐74%	31 (10.3)
75%‐100%	29 (9.6)
Provider care setting	
Inpatient only	19 (6.4)
Outpatient only	80 (27.0)
Both	197 (66.6)
Length of time the provider has been using an EHR^a^	
0‐2 years	1 (0.3)
3‐5 years	19 (6.3)
>5 years	279 (92.4)
Unknown	3 (1.0)
EHR company used most frequently	
Allscripts	29 (9.6)
Cerner	39 (13.0)
Epic	142 (47.2)
MEDITECH	18 (6.0)
Other	73 (24.2)
Number of different software systems used daily	
1	86 (28.6)
2	105 (34.9)
3	73 (24.3)
4	20 (6.6)
5 or more	17 (5.6)

a EHR: electronic health record.

**Table 2.** EHR^a^ usage before, during, and after patient visits.

Time spent in the EHR	n (%)
Prior to a patient visit	
0 minutes	5 (1.7)
1-3 minutes	62 (20.7)
3-5 minutes	95 (31.7)
5-10 minutes	88 (29.3)
>10 minutes	50 (16.7)
While in a patient visit	
0 minutes	33 (11)
1-3 minutes	82 (27.2)
3-5 minutes	79 (26.2)
5-10 minutes	65 (21.6)
>10 minutes	42 (14)
After a patient visit	
0 minutes	12 (4)
1-3 minutes	52 (17.2)
3-5 minutes	92 (30.5)
5-10 minutes	92 (30.5)
>10 minutes	54 (17.9)

a EHR: electronic health record.

### Information Elements Used for HF Decisions

Both cardiologists and general medicine physicians, across inpatient and outpatient care, consistently used medication lists, vital signs, laboratory results, diagnostic tests, problem lists, clinical notes, medical history, and allergies in their clinical practice.

**Figure 1.**
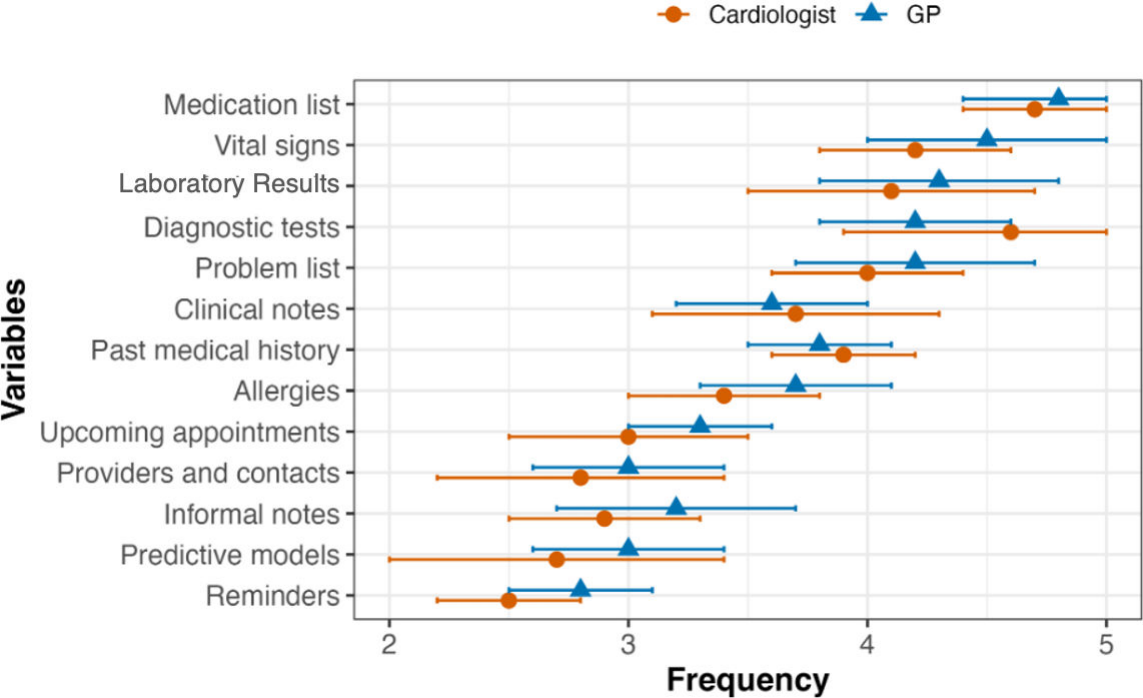
Frequency providers used clinical information in their decision-making process for inpatients. The figure illustrates the frequency of reviewing EHR data elements by cardiologists and general medicine physicians in an inpatient setting. Data elements are ordered by overall frequency of use and shown as mean (SD). EHR: electronic health record; GP: general medicine physician.

Cardiologists more frequently relied on diagnostic tests for inpatient HF treatment decisions than general medicine physicians (mean [SD] overall frequency, 4.66 [0.50] vs 4.44 [0.64]; *P*=.012). However, general medicine physicians relied on problem lists more than cardiologists (mean [SD] overall frequency, 4.63 [0.58] vs 4.43 [0.72]; *P*=.034). In contrast, there was no significant difference between both in the use of other variables (eg, medication lists [*P*=.098], vital signs [*P*=.420], and laboratory results [*P*=.244]). Figures1  2 demonstrate the clinical variables that cardiologists and general medicine physicians relied on for clinical decision-making in patients with HF.

**Figure 2.**
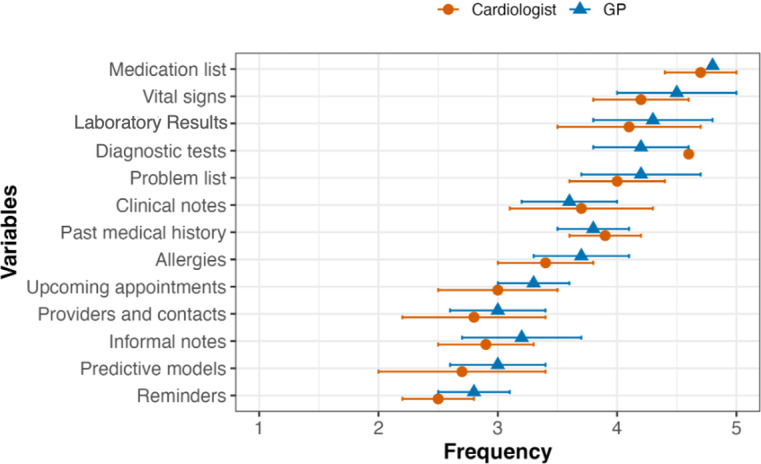
Frequency providers used clinical information in their decision-making process for outpatients. The figure illustrates the frequency of reviewing EHR data elements by cardiologists and general medicine physicians in an outpatient setting. Data elements are ordered by overall frequency of use and shown as mean (SD). EHR: electronic health record; GP: general medicine physician.

Cardiologists also relied more on diagnostic tests for outpatient treatment decisions than general medicine physicians (mean [SD] overall frequency, 4.67 [0.55] vs 4.35 [0.71]; *P*<.001). Additionally, general medicine physicians reviewed clinical notes less frequently than cardiologists (mean [SD] overall frequency, 4.50 [0.67] vs 4.65 [0.64]; *P*=.042).

### Use of Standardized Questionnaires and Predictive Models

Both provider groups underutilized standardized questionnaires and predictive models. Only 20.1% (29/144) of cardiologists and 4.5% (6/133) of general medicine physicians reported using standardized questionnaires, resulting in a significant difference (*P*<.001). Predictive model usage was similarly low, at 23.1% (33/144) among cardiologists and 27.3% (36/133) among general medicine physicians, with no significant difference between the groups (*P*=.423). The denominators (144 cardiologists and 133 general medicine physicians) reflect the subset of respondents who completed these specific survey questions; 8.3% of participants (25/302) skipped these questions.

### Time in EHR by Specialty

Cardiologists spent significantly more time reviewing the EHR before patient visits than general medicine physicians. Among cardiologists, 31.8% (47/150) spent 5‐10 minutes reviewing the EHR before patient visits, whereas 36.8% (56/152) of general medicine physicians spent only 3‐5 minutes (*P*=.035). However, there were no statistically significant differences in EHR usage during or after visits between the two groups (*P*=.247 and *P*=.170, respectively). Table 3 illustrates this in more detail.

### Number of Software Systems Used Daily

Cardiologists were more inclined to use multiple software systems, with 31.5% (47/150) using three different systems, while 40.1% (61/152) of general medicine physicians used only 2 (*P*=.001). Table 3 shows the number of different software systems used daily.

**Table 3.** Time spent in the EHR^a^ and number of software systems used daily by specialty.

Variable	Cardiologist (n=150)	General medicine physician (n=152)	*P* value
Time spent in the EHR prior to a patient visit			0.035
0 minutes	3 (2)	2 (1.3)	
1-3 minutes	26 (17.6)	36 (23.7)	
3-5 minutes	39 (26.4)	56 (36.8)	
5-10 minutes	47 (31.8)	41 (27)	
>10 minutes	33 (22.3)	17 (11.2)	
Time spent in the EHR while in a patient visit			0.247
0 minutes	14 (9.4)	19 (12.5)	
1-3 minutes	46 (30.9)	36 (23.7)	
3-5 minutes	39 (26.2)	40 (26.3)	
5-10 minutes	26 (17.5)	39 (25.7)	
>10 minutes	24 (16.1)	18 (11.8)	
Time spent in the EHR after a patient visit			0.17
0 minutes	4 (2.7)	8 (5.3)	
1-3 minutes	27 (18)	25 (16.5)	
3-5 minutes	49 (32.7)	43 (28.3)	
5-10 minutes	38 (25.3)	54 (35.5)	
>10 minutes	32 (21.3)	22 (14.5)	
The number of different software systems used on a daily basis			0.001
1	33 (22.2)	53 (34.9)	
2	44 (29.5)	61 (40.1)	
3	47 (31.5)	26 (17.1)	
4	14 (9.4)	6 (3.9)	
5 or more	11 (7.4)	6 (3.9)	

a EHR: electronic health record.

## Discussion

### Principal Findings

Both cardiologists and general medicine physicians rely heavily on specific EHR data, including medication lists, vital signs, laboratory results, diagnostic tests, problem lists, and clinical notes, when managing HF. Accordingly, new EHR interfaces should be designed to ensure these essential elements are presented in a clear, accessible format that supports timely clinical decision-making. In contrast to previous research suggesting that specialists and primary care physicians regard a patient’s clinical history—chief complaint, history of present illness, and past medical history—as among the most critical EHR sections for HF evaluation [18], our study found that both provider groups used past medical history less frequently.

### Comparison With Prior Work

While both provider groups relied on similar categories of information, our survey highlighted notable differences in how cardiologists and general medicine physicians prioritize EHR data and tools. Specifically, cardiologists placed greater emphasis on diagnostic tests, whereas general medicine physicians more frequently used the problem list. These findings are consistent with a multi-specialty survey in which 50% of specialists ranked imaging data among their top five information needs, compared with only 27% of primary care physicians [18]. Conversely, 61% of primary care physicians ranked the problem list in their top five, as opposed to just 27% of specialists [18]. This focus on the problem list aligns with the broader, long-term management responsibilities typically associated with primary care physicians. Indeed, one large study showed primary care physicians entering over 80% of all problem list items, whereas specialists contributed relatively few [19]. These differences highlight the importance of designing EHR interfaces that accommodate the distinct workflows and data needs of both specialists and general medicine physicians.

Differences also emerged in the use of software systems. Our survey suggests that cardiologists tend to use a greater number of software systems during HF care. For example, a cardiologist might navigate the primary EHR for notes and orders, a separate cardiology picture archiving and communication system for imaging [20], and device-specific platforms for pacemaker and *International Classification of Diseases* data [21]. General medicine physicians, by comparison, usually work within a single EHR ecosystem for most tasks. This describes the “network of systems” approach in specialty care: a one-size-fits-all EHR often fails to meet all specialty needs, leading many specialists to adopt “best-of-breed” solutions (multiple integrated systems tailored to their domain) [18]. In contrast, general medicine physicians may engage more with general CDSS alerts or chronic disease management prompts embedded in the EHR (eg, health maintenance reminders, drug-interaction alerts). Prior research has noted that primary care physicians place higher value on medication-related information and may be more receptive to certain decision support tied to the problem list or medication list [18].

Our survey indicates that cardiologists spend significantly more time reviewing the EHR prior to patient visits than general medicine physicians. This confirms that certain medical subspecialties experience high EHR workloads. In a large cross-specialty analysis, infectious disease, endocrinology, and nephrology were among the top specialties for total EHR time, on par with or exceeding primary care [22]. These fields, much like cardiology, manage complicated patients with multiple comorbidities and large volumes of data, which naturally translates into more time spent reviewing results, notes, and orders in the EHR [22]. Enhanced EHR design and workflow customization tailored to specialty needs may be beneficial. For example, cardiology-specific dashboards that compile recent cardiac test results, or smarter integration of hospital records and consult notes, could streamline pre-visit preparation for cardiologists. Likewise, adopting team-based planning elements in cardiology clinics (where appropriate) might offload routine data gathering from physicians. The goal would be to reduce the unnecessary time clinicians spend clicking and searching in the chart, thereby improving efficiency without sacrificing thoroughness.

Our survey revealed the underutilization of standardized questionnaires (eg, patient-reported outcome measures) and predictive risk models by both cardiologists and general medicine physicians in routine HF care. Despite the proliferation of risk prediction tools and symptom questionnaires for HF, their adoption in everyday practice remains low. Dozens of HF risk models (for outcomes such as mortality or readmission) have been published, but clinicians rarely incorporate these models at the point of care [23]. One review noted that there is no clear guideline consensus on which risk score to use, and in a large European HF registry, fewer than 1% of patients had any prognostic risk score documented in their medical record [23]. Clinicians often cite multiple barriers: predictive models developed in research may lack perceived reliability for individual patients, can be too complex or inconvenient, and may not readily fit into clinical workflows [23]. In HF, physicians may also feel that risk stratification adds little to their clinical judgment—for instance, some may perceive that patients with HF are high risk by default, so a calculated risk score might not change management [24]. This skepticism, combined with the absence of strong guideline recommendations for specific models, leads to very limited use of risk calculators at the bedside. Similarly, standardized questionnaires such as the Kansas City Cardiomyopathy Questionnaire (KCCQ) or other quality-of-life instruments are infrequently used by busy clinicians despite their proven value in research settings. Major HF guidelines encourage the assessment of patient-reported health status using tools like the KCCQ to capture symptoms and quality of life. In practice, however, the routine use of KCCQ and other patient-reported outcome surveys is rare, and these instruments are often reserved for clinical trials or specialized programs [25].

### Implications for EHR/CDSS Design

Aligned with our findings, CDSS changes should mirror observed use patterns: as cardiologists prioritized diagnostic tests, spent more pre-visit EHR time, and used more systems, the CDSS should surface diagnostic test summaries early in the workflow and aggregate test information to reduce pre-visit review and system switching; because general medicine physicians relied more on the problem list (inpatient), the CDSS should orient guidance around the problem list with clear access from inpatient views. Given that both groups consistently used medication lists, vitals, laboratory results, diagnostic tests, problem lists, and notes, decision cues should be placed adjacent to these high-traffic elements rather than in separate modules. Finally, the low reported use of standardized questionnaires and predictive models suggests placing prompts and access points for these tools on the same screens clinicians already frequent, instead of standalone locations.

### Limitations

This study has several limitations. First, the cross-sectional survey design captures only a snapshot of providers’ EHR usage patterns and priorities, limiting the ability to infer causal relationships or temporal changes. Second, data were self-reported, introducing the potential for recall and social desirability biases. Third, the survey was administered through a single vendor (Dynata), which may limit generalizability if respondents are not fully representative of all cardiologists and general medicine physicians. Fourth, patient outcomes or clinical effectiveness measures associated with EHR usage were not assessed, precluding direct links between specific EHR practices and improvements in HF care. Fifth, although the sample included providers from diverse health care systems, differences in EHR functionalities and vendors across institutions may influence how respondents interact with and prioritize EHR data. Sixth, the survey did not stratify by geographic region, institutional type, or practice setting; thus, the panel-derived sample may not perfectly reflect the national distribution of HF specialists and general medicine physicians. While specialty quotas supported planned comparisons, residual sampling bias remains possible. Seventh, although Likert scale data are ordinal, responses were treated as continuous for calculating means and conducting *t* tests. This approach, commonly used in health informatics and social science research, assumes equal intervals between response categories, which may not fully capture participants’ subjective perceptions. Results should therefore be interpreted with this methodological consideration in mind. Finally, because participants were recruited from a national US physician panel and the study team is based in the United States, the findings primarily reflect clinical practices and EHR systems in the United States. Therefore, generalizability to other countries with different health system structures and EHR implementations may be limited.

### Conclusions

Cardiologists and general medicine physicians depend on medication lists, vital signs, laboratory results, diagnostic tests, problem lists, and clinical notes to manage HF. Cardiologists place greater emphasis on diagnostic tests, spend more pre-visit EHR time, and use more software systems, whereas general medicine physicians rely more on problem lists for inpatient care. Both groups underutilize standardized questionnaires and predictive models. Tailoring the interface design of the EHR and CDSS tools to these specialty-specific needs could streamline workflows and improve HF management.

## Supplementary material

10.2196/79239 Multimedia Appendix 1 Survey questions.

## Abbreviations

CDSS: clinical decision support system
EHR: electronic health record
HF: heart failure
KCCQ: Kansas City Cardiomyopathy Questionnaire
