# Supplementary material for: Physicians’ Use of Electronic Health Record Data Elements and Decision Support Tools in Heart Failure Management: User-Centered Cross-Sectional Survey Study
Source: JMIR Cardio. 2025 Nov 14;9:e79239. doi: 10.2196/79239 (PMC12617960; doi:10.2196/79239)
Supplement: Supplementary file 1 [file cardio-v9-e79239-s001.docx]

HF Provider Survey

Start of Block: Demos

Intro This survey is part of a larger study about how electronic health records (EHR) usage affects the care of heart failure (HF) patients. The following short survey is about your use of the electronic health record (EHR) also called the electronic medical record (EMR) in the care of heart failure patients. In the survey the term EHR will be used to represent both EHR and EMR. All data collected will be kept anonymous. Please be as open as possible about your use of the EHR. 

Q44 
We care about the quality of our survey data and hope to receive the most accurate measures of your opinions, so it is important to us that you thoughtfully provide your best answer to each question in the survey.

Do you commit to providing your thoughtful and honest answers to the questions in this survey?

- I will provide my best answers  (1) 

- I will not provide my best answers  (4) 

- I can't commit either way  (5) 

Skip To: End of Block If We care about the quality of our survey data and hope to receive the most accurate measures of yo... != I will provide my best answers

| Page Break |  |
| --- | --- |

|  |
| --- |

Q43 In which country do you currently reside?

▼ Afghanistan (1) ... Zimbabwe (1357)

Skip To: End of Block If List of Countries != United States of America

Demo 1 What is your clinical role? Check the one that applies most closely to your role.

- Internal Medicine Physician  (7) 

- Family Medicine Physician  (6) 

- Heart Failure Cardiologist  (9) 

- Cardiologist  (1) 

- Other  (8) ________________________________________________

| Page Break |  |
| --- | --- |

Demo 2 Do you care for patients diagnosed with heart failure?

- Yes  (1) 

- No  (2) 

End of Block: Demos

Start of Block: Default Question Block

Demo 2.1 What % of your time do you spend caring for heart failure patients?

- 0%  (1) 

- 1-24%  (2) 

- 25-49%  (3) 

- 50-74%  (5) 

- 75%-100%  (6) 

Demo 3 Do you care for inpatients, outpatients, or both?

- Inpatient only  (1) 

- Outpatient only  (2) 

- Both  (3) 

| Page Break |  |
| --- | --- |

EHR 1 For how long have you been using an electronic health record (EHR) system?

- 0-2 years  (1) 

- 3-5 years  (2) 

- >5 years  (3) 

- I don't use an EHR  (4) 

EHR 2 For an individual patient, how much time do you spend using the EHR to review patient data ***prior*** to seeing the patient?

- 0 minutes  (1) 

- 1-3 minutes  (2) 

- 3-5 minutes  (3) 

- 5-10 minutes  (4) 

- > 10 minutes  (5) 

EHR 3 For an individual patient, how much time do you spend using the EHR to review patient data ***while*** seeing the patient in the exam room?

- 0 minutes  (1) 

- 1-3 minutes  (2) 

- 3-5 minutes  (3) 

- 5-10 minutes  (4) 

- > 10 minutes  (5) 

EHR 4 For an individual patient, how much time do you spend using the EHR to make treatment decisions after seeing the patient?

- 0 minutes  (1) 

- 1-3 minutes  (2) 

- 3-5 minutes  (3) 

- 5-10 minutes  (4) 

- > 10 minutes  (5) 

| Page Break |  |
| --- | --- |

EHR 5 How many different types of software systems do you use on a daily basis to prepare for and/or document patient interactions? For example, a lab system to view labs and a radiology system to view chest X-rays would be 2 different software systems.

- 1  (1) 

- 2  (2) 

- 3  (3) 

- 4  (4) 

- 5 or more  (5) 

EHR 6 What is the name of the EHR company that supplies the EHR you use most frequently?

- Epic  (4) 

- Cerner  (5) 

- MEDITECH  (6) 

- Evident  (7) 

- Allscripts  (8) 

- Other  (9) ________________________________________________

EHR 7 Complete the sentence: "Using the electronic health record (EHR) system to make patient care decisions is..."

________________________________________________________________

| Page Break |  |
| --- | --- |

Display This Question:

If Do you care for inpatients, outpatients, or both? = Inpatient only

Or Do you care for inpatients, outpatients, or both? = Both

|  |
| --- |

Treatment 1.1 Out of the following list of information on an electronic health record (EHR) system, how frequently do you use the following information for heart failure patient treatment decisions for ***inpatients***?

|  | Never (1) | Rarely (2) | Sometimes (3) | Frequently (4) | Always (5) |
| --- | --- | --- | --- | --- | --- |
| Lab results (e.g. Na, K, BUN, SCr) (1) |  |  |  |  |  |
| Diagnostic tests (Cardiac MRI, Echo, Cardiac Cath, etc.) (2) |  |  |  |  |  |
| Predictive models or risk stratification models (3) |  |  |  |  |  |
| Past medical history (e.g. CAD, DM2, HTN, etc.) (4) |  |  |  |  |  |
| Problem list (Active medical problems) (5) |  |  |  |  |  |
| Allergies (6) |  |  |  |  |  |
| Clinical notes (formal documentation) (7) |  |  |  |  |  |
| Vital signs (e.g. heart rate, blood pressure, etc.) (8) |  |  |  |  |  |
| Upcoming appointments (9) |  |  |  |  |  |
| Providers and contacts (10) |  |  |  |  |  |
| Medication list (11) |  |  |  |  |  |
| Reminders or sticky notes (i.e. information about the patient for personal reference) (12) |  |  |  |  |  |
| Informal notes to other providers (i.e. notes and comments from one provider to another) (13) |  |  |  |  |  |

| Page Break |  |
| --- | --- |

Display This Question:

If Do you care for inpatients, outpatients, or both? = Outpatient only

Or Do you care for inpatients, outpatients, or both? = Both

|  |
| --- |

Treatment 1.2 Out of the following list of information on an electronic health record (EHR) system, how frequently do you use the following information for heart failure patient treatment decisions for ***outp***atients?

|  | Never (1) | Rarely (2) | Sometimes (3) | Frequently (4) | Always (5) |
| --- | --- | --- | --- | --- | --- |
| Lab results (e.g. Na, K, BUN, SCr) (1) |  |  |  |  |  |
| Diagnostic tests (Cardiac MRI, Echo, Cardiac Cath, etc.) (2) |  |  |  |  |  |
| Predictive models or risk stratification models (3) |  |  |  |  |  |
| Past medical history (e.g. CAD, DM2, HTN, etc.) (4) |  |  |  |  |  |
| Problem list (Active medical problems) (5) |  |  |  |  |  |
| Allergies (6) |  |  |  |  |  |
| Clinical notes (formal documentation) (7) |  |  |  |  |  |
| Vital signs (e.g. heart rate, blood pressure, etc.) (8) |  |  |  |  |  |
| Upcoming appointments (9) |  |  |  |  |  |
| Providers and contacts (10) |  |  |  |  |  |
| Medication list (11) |  |  |  |  |  |
| Reminders or sticky notes (i.e. information about the patient for personal reference) (12) |  |  |  |  |  |
| Informal notes to other providers (i.e. notes and comments from one provider to another) (13) |  |  |  |  |  |

| Page Break |  |
| --- | --- |

Display This Question:

If Do you care for inpatients, outpatients, or both? = Inpatient only

Or Do you care for inpatients, outpatients, or both? = Both

Treatment 2.1 What are the top three pieces of data (in order of importance) that you initially look for when making treatment decisions for heart failure ***inpatients***?

________________________________________________________________

________________________________________________________________

________________________________________________________________

________________________________________________________________

________________________________________________________________

Display This Question:

If Do you care for inpatients, outpatients, or both? = Outpatient only

Or Do you care for inpatients, outpatients, or both? = Both

Treatment 2.2 What are the top three pieces of data (in order of importance) that you initially look for when making treatment decisions for heart failure ***outpatients***?

________________________________________________________________

________________________________________________________________

________________________________________________________________

________________________________________________________________

________________________________________________________________

| Page Break |  |
| --- | --- |

Display This Question:

If Do you care for inpatients, outpatients, or both? = Outpatient only

Or Do you care for inpatients, outpatients, or both? = Both

Treatment 3 Do you use a standardized patient questionnaire to help manage outpatients? For example, the Kansas City Cardiomyopathy Questionnaire.

▼ Yes (1) ... No (8)

Display This Question:

If Do you use a standardized patient questionnaire to help manage outpatients? For example, the Kans... = Yes

Treatment 3.1 What standardized patient questionnaire do you use?

________________________________________________________________

Display This Question:

If Do you use a standardized patient questionnaire to help manage outpatients? For example, the Kans... = No

Treatment 3.2 If you do not use a standardized patient questionnaire, why not?

________________________________________________________________

| Page Break |  |
| --- | --- |

Display This Question:

If Do you care for inpatients, outpatients, or both? = Outpatient only

Or Do you care for inpatients, outpatients, or both? = Both

Treatment 4 Do you use a predictive model or risk stratification system to help manage outpatients? For example, the Seattle HF Model.

▼ Yes (1) ... No (2)

Display This Question:

If Do you use a predictive model or risk stratification system to help manage outpatients? For examp... = Yes

Treatment 4.1 What predictive model or risk stratification system do you use to help manage outpatients?

________________________________________________________________

Display This Question:

If Do you use a predictive model or risk stratification system to help manage outpatients? For examp... = Yes

Treatment 4.2 How frequently do you use the predictive model or risk stratification system to make a treatment decision?

- Not at all  (1) 

- Less than once a week  (2) 

- At least once a week  (3) 

- About once a day  (4) 

- Many times a day  (5) 

Display This Question:

If Do you use a predictive model or risk stratification system to help manage outpatients? For examp... = No

Treatment 4.3 If you don't use a predictive model or risk stratification system, why not?

________________________________________________________________

| Page Break |  |
| --- | --- |

Treatment 5 Aside from what is currently available in your primary electronic health record (EHR), what additional data or information might help you to make treatment decisions for heart failure patients and why?

________________________________________________________________

________________________________________________________________

________________________________________________________________

________________________________________________________________

________________________________________________________________

End of Block: Default Question Block
